# Identifying Subpopulations with Distinct Response to Treatment Using Plasma Biomarkers in Acute Heart Failure: Results from the PROTECT Trial: Differential Response in Acute Heart Failure

**DOI:** 10.1007/s10557-017-6726-1

**Published:** 2017-06-27

**Authors:** Licette C. Y. Liu, Mattia A. E. Valente, Douwe Postmus, Christopher M. O’Connor, Marco Metra, Howard C. Dittrich, Piotr Ponikowski, John R. Teerlink, Gad Cotter, Beth Davison, John G. F. Cleland, Michael M. Givertz, Daniel M. Bloomfield, Dirk J. van Veldhuisen, Hans L. Hillege, Peter van der Meer, Adriaan A. Voors

**Affiliations:** 1 Department of Cardiology, University of Groningen, University Medical Center Groningen, Groningen, The Netherlands; 2 Department of Epidemiology, University of Groningen, University Medical Center Groningen, Groningen, The Netherlands; 3 Inova Heart and Vascular Institute, Falls Church, VA USA; 4 University of Brescia, Brescia, Italy; 5 Cardiovascular Research Center, University of Iowa Carver College of Medicine, Iowa City, IA USA; 6 Medical University, Clinical Military Hospital, Wroclaw, Poland; 7 University of California at San Francisco and San Francisco Veterans Affairs Medical Center, San Francisco, CA USA; 8 Momentum Research, Durham, NC USA; 9 Imperial College, London, UK; 10 Harvard Medical School, Brigham and Women’s Hospital, Boston, MA USA; 11 Merck Research Laboratories, Rahway, NJ USA

**Keywords:** Acute heart failure, Treatment heterogeneity, Biomarkers, Subpopulation treatment effect pattern plot, Rolofylline

## Abstract

**Background:**

Over the last 50 years, clinical trials of novel interventions for acute heart failure (AHF) have, with few exceptions, been neutral or shown harm. We hypothesize that this might be related to a differential response to pharmacological therapy.

**Methods:**

We studied the magnitude of treatment effect of rolofylline across clinical characteristics and plasma biomarkers in 2033 AHF patients and derived a biomarker-based responder sum score model. Treatment response was survival from all-cause mortality through day 180.

**Results:**

In the overall study population, rolofylline had no effect on mortality (HR 1.03, 95% CI 0.82–1.28, *p* = 0.808). We found no treatment interaction across clinical characteristics, but we found interactions between several biomarkers and rolofylline. The biomarker-based sum score model included TNF-R1α, ST2, WAP four-disulfide core domain protein HE4 (WAP-4C), and total cholesterol, and the score ranged between 0 and 4. In patients with score 4 (those with increased TNF-R1α, ST2, WAP-4C, and low total cholesterol), treatment with rolofylline was beneficial (HR 0.61, 95% CI 0.40–0.92, *p* = 0.019). In patients with score 0, treatment with rolofylline was harmful (HR 5.52, 95% CI 1.68–18.13, *p* = 0.005; treatment by score interaction *p* < 0.001). Internal validation estimated similar hazard ratio estimates (0 points: HR 5.56, 95% CI 5.27–7–5.87; 1 point: HR 1.31, 95% CI 1.25–1.33; 2 points: HR 0.75, 95% CI 0.74–0.76; 3 points: HR 1.13, 95% CI 1.11–1.15; 4 points, HR 0.61, 95% CI 0.61–0.62) compared to the original data.

**Conclusion:**

Biomarkers are superior to clinical characteristics to study treatment heterogeneity in acute heart failure.

**Electronic supplementary material:**

The online version of this article (doi:10.1007/s10557-017-6726-1) contains supplementary material, which is available to authorized users.

## Introduction

Acute heart failure is one of the leading causes of hospitalization among patients ≥65 years and carries a dismal prognosis and high economic burden [1, 2]. Treatment of acute heart failure remains a therapeutic challenge as current guideline recommendations are based on smaller, nonrandomized trials and expert opinion [2]. Many potential interventions for acute heart failure have been studied, but most of them have failed to improve prognosis and none of them have entered guideline recommendations [2–8]. Several reasons have been addressed: the wrong drugs might have been studied, or the wrong study designs might have been used. We hypothesize that one of the most important explanations is likely the heterogeneity of patients with acute heart failure. In clinical trials, treatment arms are analyzed as a whole and there seems to be some consensus that heterogeneity of the relative treatment effect is rare. However, in a trial including relatively unselected heart failure patients, differential responses to treatment are to be expected. The wide variety in the underlying pathophysiological mechanisms and clinical presentations in acute heart failure challenges selection of an appropriate study population and, intuitively, hampers a universal treatment that fits all patients. Based on these factors, acute heart failure might hold a great potential for precision medicine. Understanding differences in treatment response is imperative to customize therapy. Subgroup analyses are thus necessary to identify potential interactions between underlying pathophysiologies and treatment effect. However, conventional subgroup analysis based on clinical characteristics often failed to demonstrate any interaction effect with treatment. As plasma biomarkers may characterize individual involved pathophysiological pathways, these markers can be particularly useful to study variation in drug response profiles. In the present study, we propose a novel method to use a large panel of established and novel emerging biomarkers to distinguish responders from nonresponders to pharmacological therapy in acute heart failure.

## Methods

### Patient Population

We investigated patients enrolled in the Placebo-controlled Randomized Study of Selective A1 Adenosine Receptor Antagonist Rolofylline for Patients Hospitalized with Acute Decompensated Heart Failure and Volume Overload to Assess Treatment Effect on Congestion and Renal Function (PROTECT) trial [7, 9]. The design and main results of the study have been published elsewhere [7, 9]. Between May 2007 and January 2009, 2033 acute heart failure patients were randomized to receive 30 mg rolofylline or placebo administered as a daily 4 h infusion for 3 days in a 2:1 ratio. Briefly, eligible patients were male or female, ≥18 years of age, hospitalized for signs and symptoms of acute heart failure, defined as persistent dyspnea at rest or minimal exertion and fluid overload requiring intravenous loop-diuretic therapy, with impaired renal function (estimated creatinine clearance of 20–80 mL/min calculated by the Cockcroft-Gault equation (corrected for height in edematous or obese patients ≥100 kg)) and elevated natriuretic peptide levels (brain natriuretic peptide (BNP) or N-terminal pro-BNP levels ≥500 or ≥2000 pg/mL, respectively). Key exclusion criteria included systolic blood pressure <90 or ≥160 mmHg, acute coronary syndrome and treatment with positive inotropic agents, vasopressors or vasodilatators (with the exception of intravenous nitrates), ultrafiltration, hemofiltration, dialysis, or mechanical support. The PROTECT trial conformed to the Declaration of Helsinki, local ethics committees at each participating center approved the study, and all participating patients provided written informed consent. Demographic, clinical, and biochemical variables of interest were collected within the framework of the PROTECT study. The PROTECT trial was registered at ClinicalTrials.gov: NCT00328692 and NCT00354458.

### Biochemical Analyses

Venous blood sampling was performed daily through day 6 or discharge and day 7 and 14 with K_3_EDTA Vacutainers. For the present analysis, we analyzed baseline values. We retrospectively used a panel of 48 markers that were already available and published before [10]. These biomarkers were a combination of routine laboratory markers from local laboratories, as well as centrally measured biomarkers, and there was no specific selection for the present study. The following biomarkers were available: albumin, alanine transaminase, aspartate transaminase, bicarbonate, blood urea nitrogen (BUN), chloride, creatinine, glucose, hemoglobin, platelet count, potassium, red blood cell count, sodium, total cholesterol, triglycerides, uric acid, and white blood cell count were measured in a central laboratory (ICON Laboratories, Farmingdale, NY). Remaining plasma was then separated from blood as soon as possible by centrifugation at 1500×*g* for 10 min and stored at −80 °C. Thirty-one biomarkers of different biological domains (inflammation, oxidative stress, remodeling, cardiomyocyte stretch, angiogenesis, atherosclerosis, renal function) were determined immediately after defrosting plasma. The following biomarkers were determined: galectin-3, myeloperoxidase, and neutrophil gelatinase-associated lipocalin were measured using sandwich enzyme-linked immonsorbent assays (ELISA) on a microtiter plate; angiogenin and C-reactive protein were measured using competitive ELISAs on a Luminex® platform; D-dimer, endothelial cell-selective adhesion molecule (ESAM), growth differentiation factor 15 (GDF-15), lymphotoxin beta receptor, mesothelin, neuropilin, N-terminal pro C-type natriuretic peptide, osteopontin, procalcitonin, pentraxin-3, periostin, polymeric immunoglobulin receptor, pro-adrenomedullin, prosaposin B, receptor for advanced glycation endproducts, soluble ST-2 (ST2), syndecan-1, tumor necrosis factor alpha receptor 1 (TNF-R1α), tumor necrosis factor receptor superfamily member, vascular endothelial growth receptor 1, and WAP four-disulfide core domain protein HE4 (WAP-4C) were measured using sandwich ELISAs on a Luminex® platform (Alere™ Inc., San Diego, CA, USA). Immunoassays for procalcitonin, proADM, galectin-3, and ST2 were developed by Alere™. These research assays have not been standardized to the commercialized assays used in research or in clinical use. Further, the extent to which each Alere™ assay correlates with the commercial assay is not fully characterized. Kidney Injury Molecule 1, BNP, interleukin-6, endothelin-1, and cardiac-specific Troponin I were measured in frozen plasma samples using highly sensitive single molecule counting (SMC™) technology (RUO, Erenna® Immunoassay System, Singulex Inc., Alameda, CA, USA).

#### Statistical Analysis

Continuous variables are summarized as mean ± standard deviation if normally distributed or median and interquartile range if nonnormally distributed. Categorical variables are reported as percentages of observations. Analysis of variance, Student’s *t* test, Chi-square test, and Mann-Whitney *U* test were used as appropriate for group comparisons. We included all 2033 patients enrolled in the PROTECT trial for the present analysis. Patients with missing baseline values of the covariate of interest were excluded from the analysis of the subgroup of interest. A full, complete 48-biomarker panel was obtained for 1266 patients. Treatment response was defined as survival from all-cause mortality through day 180. We explored treatment heterogeneity across clinical characteristics by examining forest plots. Subgroups based on clinical characteristics were defined by median (if continuous) or by category (if categorical). Interaction was estimated by Cox proportional hazard analysis using an interaction term. We explored treatment heterogeneity across the levels of biomarkers using forest plots. Subgroups based on biomarkers were defined by median of the biomarker of interest. Also, to explore treatment heterogeneity across the spectrum of the biomarkers and to establish a clinical relevant cut-point, we also studied differential response across the spectrum of biomarkers using the subpopulation treatment effect pattern plot (STEPP) [11, 12]. A *p* value of ≤0.05 was considered statistically significant. All analyses were performed using R: A Language and Environment for Statistical Computing, version 3.1.1 (R Foundation for Statistical Computing, Vienna, Austria). The *stepp* package was used to perform STEPP analyses.

### Subpopulation Treatment Effect Pattern Plot

STEPP is a novel graphical method and allows exploring differential treatment effect across the continuum of a biomarker [11, 12]. STEPP divides the overall study population into overlapping subgroups defined by the median value. Treatment effect will be assessed in each subpopulation. By creating overlapping subpopulations, STEPP is a more accurate, robust, and reliable statistical method to explore treatment heterogeneity across subgroups. The advantage of this approach compared with traditional analysis is that STEPP greatly increases the precision of the estimated treatment effect, improves statistical power to detect treatment effect heterogeneity, and reduces the chance of false-negative discovery. In addition, STEPP allows studying differential response across the continuum of a variable. In our study, overlapping subgroups were generated so that the maximum size of each subpopulation was set between 150 and 300 patients, while the number of patients belonging to adjacent subgroups was 50–150. STEPP depicts estimates of absolute risk and relative risk across subpopulations with increasing median concentration levels of the biomarker of interest. STEPP generates three plots: effect estimated of the two treatments against the median of each subpopulation, effect differences of the two treatments in absolute scale against the median of each subpopulation, and effect ratios of the two treatments in relative scale against the median of each subpopulation. To evaluate the statistical significance of observed response differences, permutation tests are performed. In our analysis, the number of permutation tests was set at 2500. To conclude that there is a significant interaction with treatment, the plots should show a divergent and consistent pattern along the continuum of the biomarker.

### Sum Score Model

To identify subgroups with distinct response to treatment, we developed a score model. For this model, we selected biomarkers only. Because STEPP allowed us to study treatment heterogeneity across the continuum of a biomarker, we explored each STEPP plot of the relative treatment effect. Only biomarkers with both a significant treatment interaction and a divergent and consistent pattern plot were selected. The STEPP plot was used to determine clinically relevant cut-points: the concentration where the STEPP plot demonstrated a cross-over in relative treatment effect. We created a new factor variable and divided patients into subgroups according to this cut-point. The new factor variables of biomarkers showing a significant treatment interaction were entered in a multivariable Cox regression model. The scores were related to the values of the predictors; thus, the scores were directly derived from the regression coefficients, by multiplying by 2 and rounding them. Thus, only biomarkers with a *β*-coefficient > 0.25 were included in the final sum score model. Treatment effect was then estimated for each subgroup of possible value of the score.

### Internal Validation

To test the robustness of our model results, we performed superpopulation bootstrap with 1000 iterations. We created a superpopulation by multiplying each subject ten times. From the created superpopulation, we sampled 1000 times a resample without replacement with the same sampling fraction as the original sample (*n* = 2033). We tested the treatment effect in each resample and estimates of hazard ratios and 95% CIs around the variable’s coefficients from each subgroup were compared with those from the original population.

## Results

**Fig. 1:**
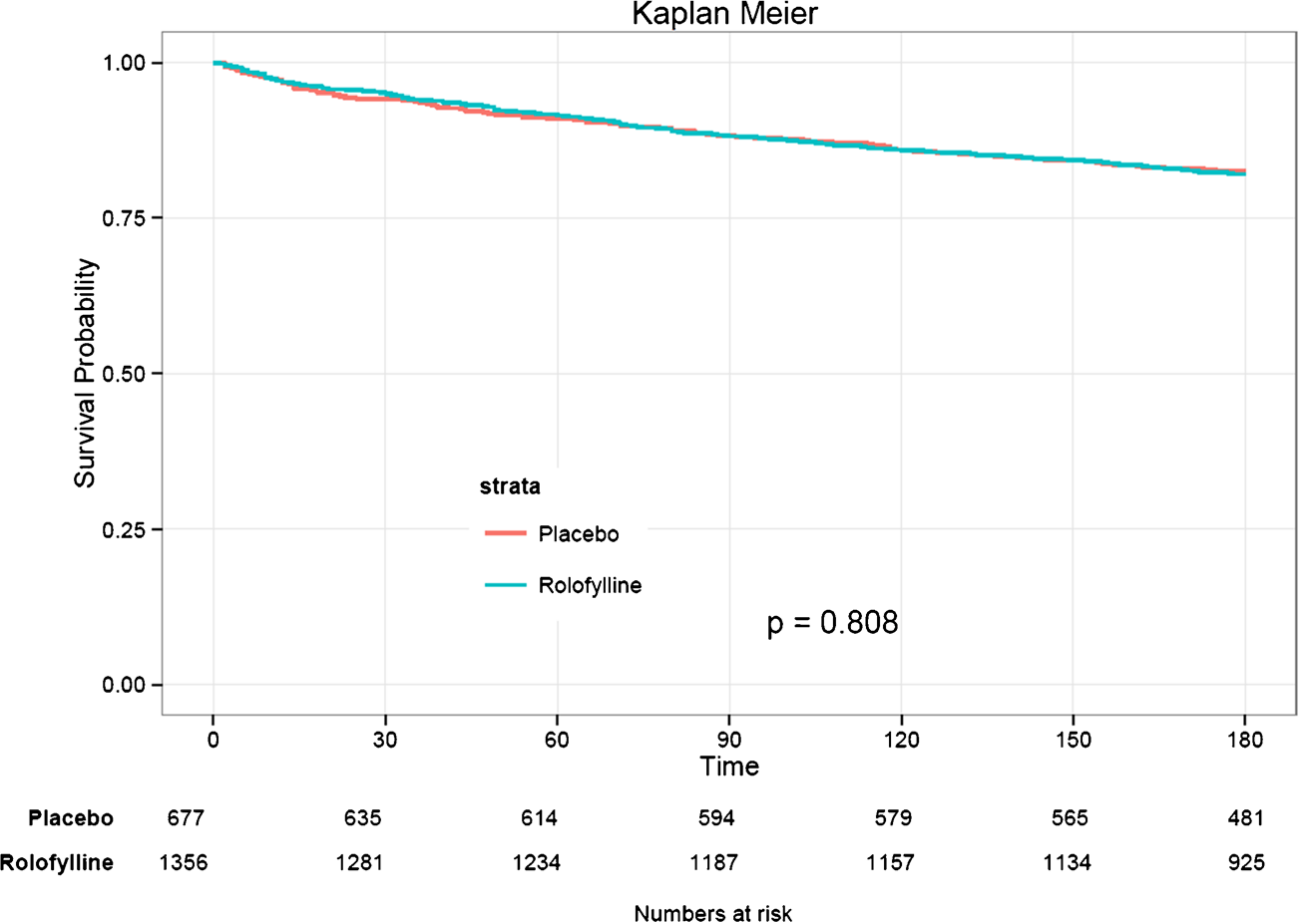
Kaplan-Meier curve for 180-day all-cause mortality in the overall study population

Baseline characteristics of the subpopulation of patients with at least one biomarker measured are presented in Table 1. Mean age of the patients was 70.2 ± 11.6 years and 67% were male. The majority of patients had ischemic heart disease and hypertension and used renin-angiotensin-aldosterone system blockers and/or beta-blockers. Mean left ventricular ejection fraction was 32.2 ± 13%, and blood pressure was well-controlled (124.4 ± 17.6/73.9 ± 11.8 mmHg). No pronounced differences were observed for patient characteristics according to treatment arm. In the total study population, treatment with rolofylline did not result in a decreased risk for all-cause death through day 180 (HR 1.03, 95% CI 0.82–1.28, *p* = 0.808, Fig. 1).

**Table 1:** Baseline characteristics according to treatment

Variable	Rolofylline	Placebo	*p* value
Number	1313	650	
Demographics			
Sex (% male)	66.9 (878)	66.6 (433)	0.951
Age (years)	70.1 ± 11.7	70.2 ± 11.5	0.852
BMI (kg/m^2^)	28.9 ± 6.1	28.7 ± 6.2	0.612
LVEF (%)	32.1 ± 12.7	32.4 ± 13.6	0.709
HFPEF (%)	18.6 (118)	21 (65)	0.447
Systolic blood pressure (mmHg)	124.4 ± 17.5	124.3 ± 17.8	0.911
Diastolic blood pressure (mmHg)	73.7 ± 11.8	74.1 ± 11.9	0.472
Heart rate (beats/min)	80 ± 15.3	80.6 ± 15.7	0.408
Clinical profile			
Atrial fibrillation on presentation	38.1 (203)	47.7 (124)	0.012
Orthopnea (%)	95.8 (1242)	96.9 (622)	0.312
Rales (%)	63 (826)	58.3 (378)	0.051
Edema (%)	67.8 (890)	67.4 (438)	0.881
Jugular venous pressure (%)	40.8 (485)	41.4 (242)	0.867
Medical history			
Hypertension (%)	80.3 (1054)	77.4 (503)	0.153
Diabetes mellitus (%)	45.2 (593)	46.2 (300)	0.725
Hypercholesterolemia (%)	51.2 (672)	52.1 (338)	0.744
Smoking (%)	21.3 (279)	19 (123)	0.25
Ischemic heart disease (%)	70.4 (923)	67.8 (440)	0.259
Myocardial infarction (%)	51 (668)	46.4 (301)	0.059
PCI (%)	26.4 (343)	25.3 (163)	0.671
CABG (%)	21.2 (276)	21.8 (140)	0.804
Peripheral vascular disease (%)	11.4 (149)	9.6 (62)	0.256
Atrial fibrillation (%)	53.2 (696)	57 (366)	0.125
NYHA class			0.18
I/II	15.8 (208)	19.2 (125)	
II	48.9 (642)	46.9 (305)	
IV	29.9 (393)	28.9 (188)	
ICD therapy (%)	16.1 (212)	15.3 (99)	0.658
CRT therapy (%)	10.2 (134)	9.6 (62)	0.717
Stroke (%)	9 (118)	9.2 (60)	0.926
COPD (%)	19.9 (261)	19.8 (128)	0.983
Prior medication use			
ACE inhibitors or ARB (%)	76.2 (1000)	74.6 (485)	0.469
Beta blockers (%)	76.7 (1006)	76.5 (497)	0.961
Mineralocorticoid receptor antagonists (%)	44.6 (585)	43.4 (282)	0.648
Calcium antagonists (%)	15 (196)	10.6 (69)	0.01
Nitrates (%)	26.9 (353)	23.6 (153)	0.126
Digoxin (%)	27.5 (361)	30.3 (197)	0.216
Laboratory values			
Creatinine (mg/dL)	1.4 [1.1–1.8]	1.3 [1.1–1.7]	0.135
Creatinine clearance (mL/min)	48.6 [36.4–62.9]	49.8 [37.8–64.9]	0.16
Blood urea nitrogen (mg/dL)	30 [22–41]	29 [22–41]	0.303
Sodium (mmol/L)	140 [137–142]	140 [137–142]	0.265
Potassium (mmol/L)	4.2 [3.9–4.7]	4.2 [3.9–4.6]	0.64
Hemoglobin (g/dL)	12.7 ± 2	12.6 ± 1.9	0.527
Anemia (%)	42 (487)	43.5 (254)	0.592
Total cholesterol (mmol/L)	3.8 ± 1.1	3.8 ± 1.1	0.59
Triglycerides (mmol/L)	101.3 ± 54.1	101.9 ± 57.9	0.813
BNP (pg/mL)	442.8 [250.5–789.9]	458.6 [259.5–829.3]	0.537

### Treatment Heterogeneity of Rolofylline

#### Clinical Characteristics

**Fig. 2:**
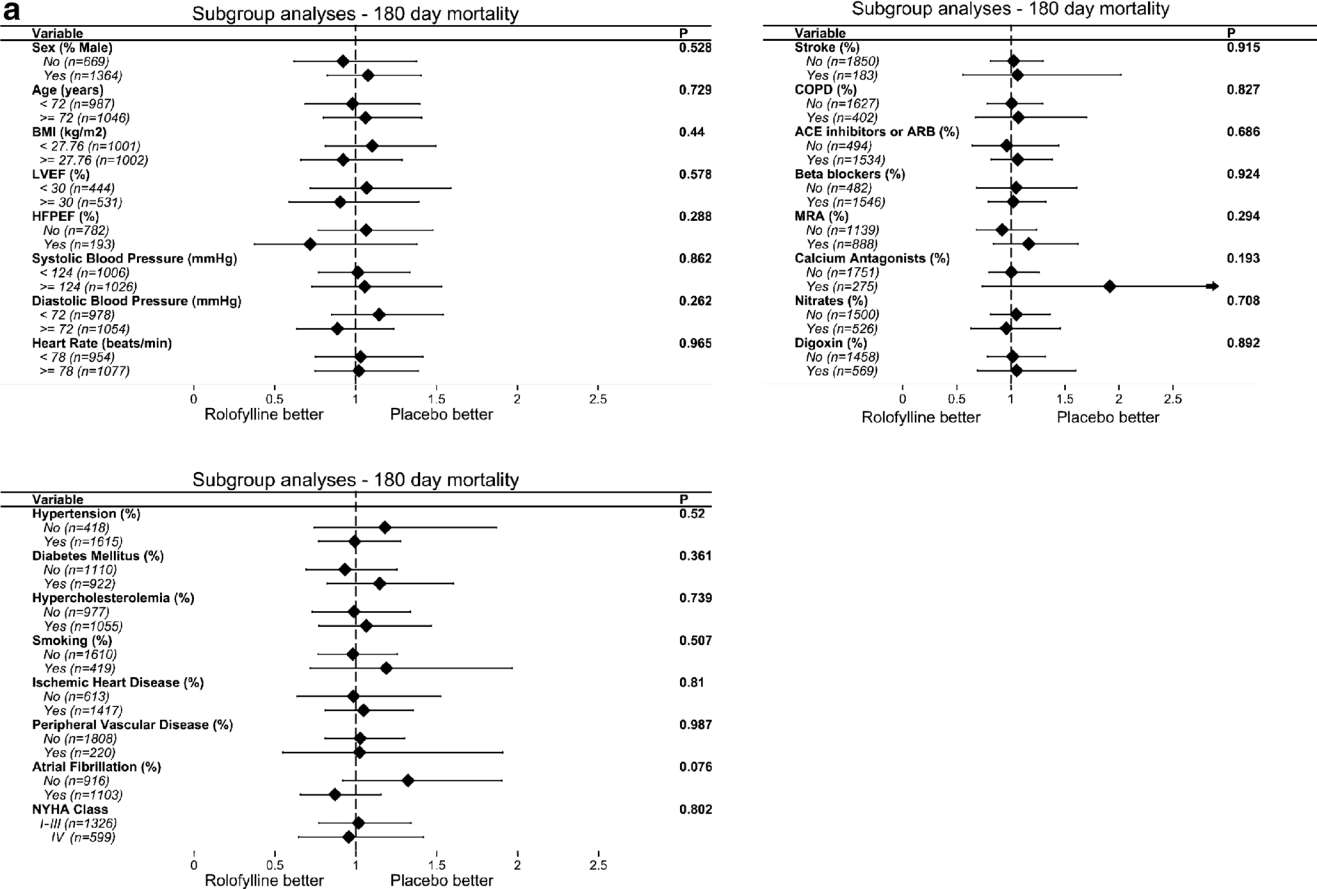

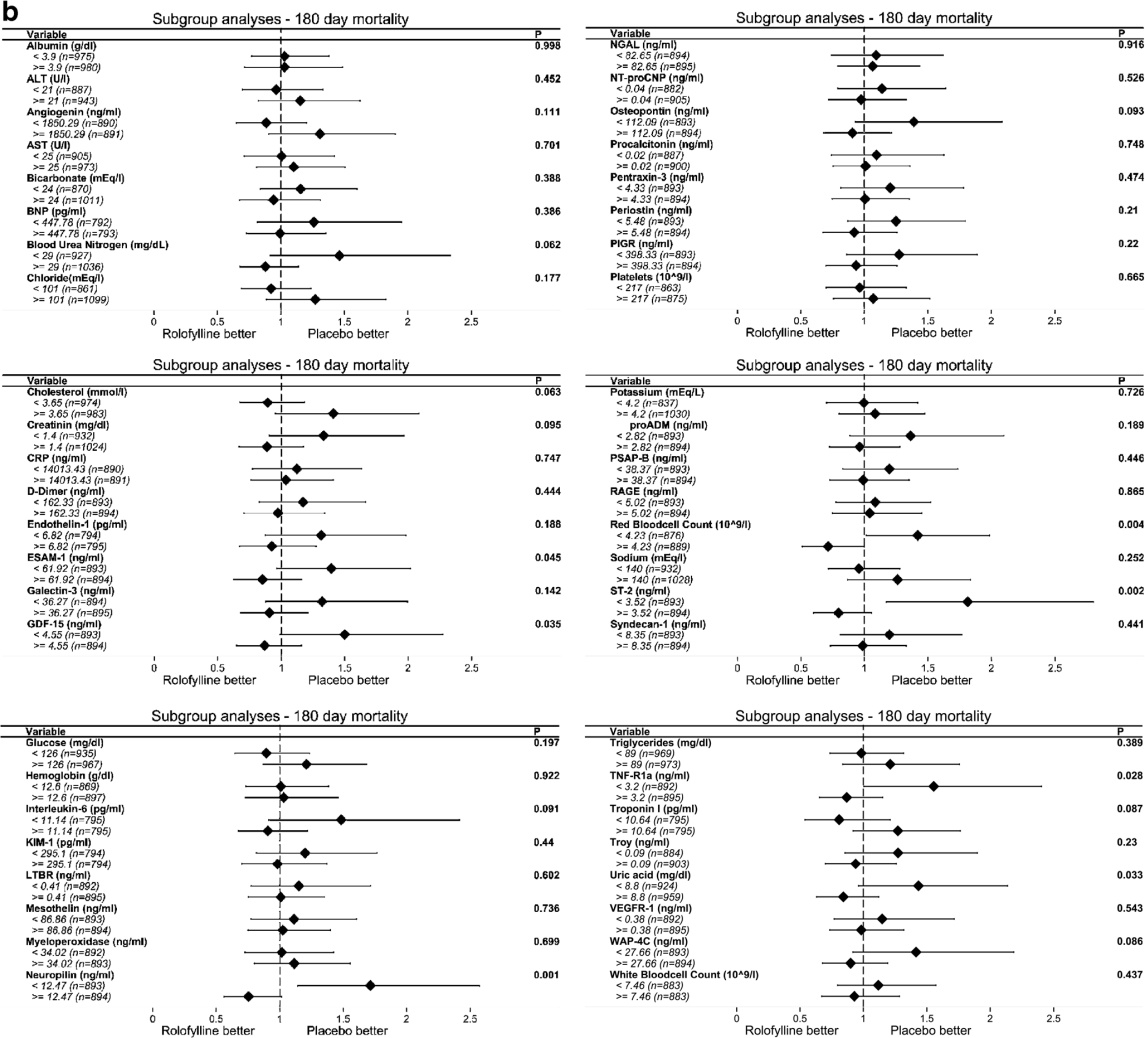
**a** Forest plots of the treatment effect for 180-day all-cause mortality per clinical characteristic. Subgroups were defined by median (if continuous) or by category (if categorical). No significant interactions with treatment were observed for subgroups defined by clinical characteristics. Abbreviations: *BMI*, body mass index; *LVEF*, left ventricular ejection fraction; *HFPEF*, heart failure with preserved ejection fraction; *COPD*, chronic obstructive pulmonary disease; *ACE*, angiotensin converting enzyme; *ARB*, angiotensin receptor blocker; *MRA*, mineralocorticoid receptor antagonist. **b** Forest plots of the treatment effect for 180-day all-cause mortality per biomarker level. Subgroups were defined by median. Forest plots demonstrated significant treatment interactions with *ESAM* (*p* = 0.045), *GDF-15* (*p* = 0.035), neuropilin (*p* = 0.001), red blood cell count (*p* = 0.004), ST2 (*p* = 0.002), *TNF-R1α* (*p* = 0.028), and uric acid (*p* = 0.033). Abbreviations: *ANP*, atrial natriuretic peptide; *BNP*, brain natriuretic peptide; *CRP*, C-reactive protein; *ESAM*, endothelial cell selective adhesion molecule; *GDF-15*, growth differentation factor; *LTBR*, lymphotoxin beta receptor; *NGAL*, neutrophil gelatinase-associated lipocalin; *NT-proCNP*, N-terminal pro-C-type natriuretic peptide; *PIGR*, polymeric immunoglobulin receptor; *proADM*, pro-adrenomedullin; *PSAPB*, prosaposin B; *RAGE*, receptor for advanced glycation endproducts; *TNF-R1a*, tumor necrosis factor α receptor-1; *TROY*, tumor necrosis factor receptor superfamily member; *TnI*, troponine I; *ET-1*, endothelin 1; *IL-6*, interleukin 6

Subgroup analyses across clinical characteristics are presented in Fig. 2a. We found no interactions between treatment and age, sex, and left ventricular ejection fraction (LVEF). Also, we found no interactions between treatment and risk factors, such as diabetes mellitus, hypertension, hypercholesterolemia, and smoking, or medical history, such as ischemic heart disease or atrial fibrillation. Also, no significant treatment interaction with medication was observed.

#### Biomarkers

Subgroup analyses across patient groups divided by the median of the biomarker of interest are summarized in Fig. 2b. In general, we observed a greater treatment benefit of rolofylline in patients with elevated levels of most of the biomarkers. We found significant interactions between treatment and ESAM (*p* = 0.045), GDF-15 (*p* = 0.035), neuropilin (*p* = 0.001), red blood cell count (*p* = 0.004), ST2 (*p* = 0.002), TNF-R1α (*p* = 0.028), and uric acid (*p* = 0.033) (Fig. 2b), for which rolofylline had a greater treatment effect in subgroups above the median of the biomarker of interest.

**Fig. 3:**
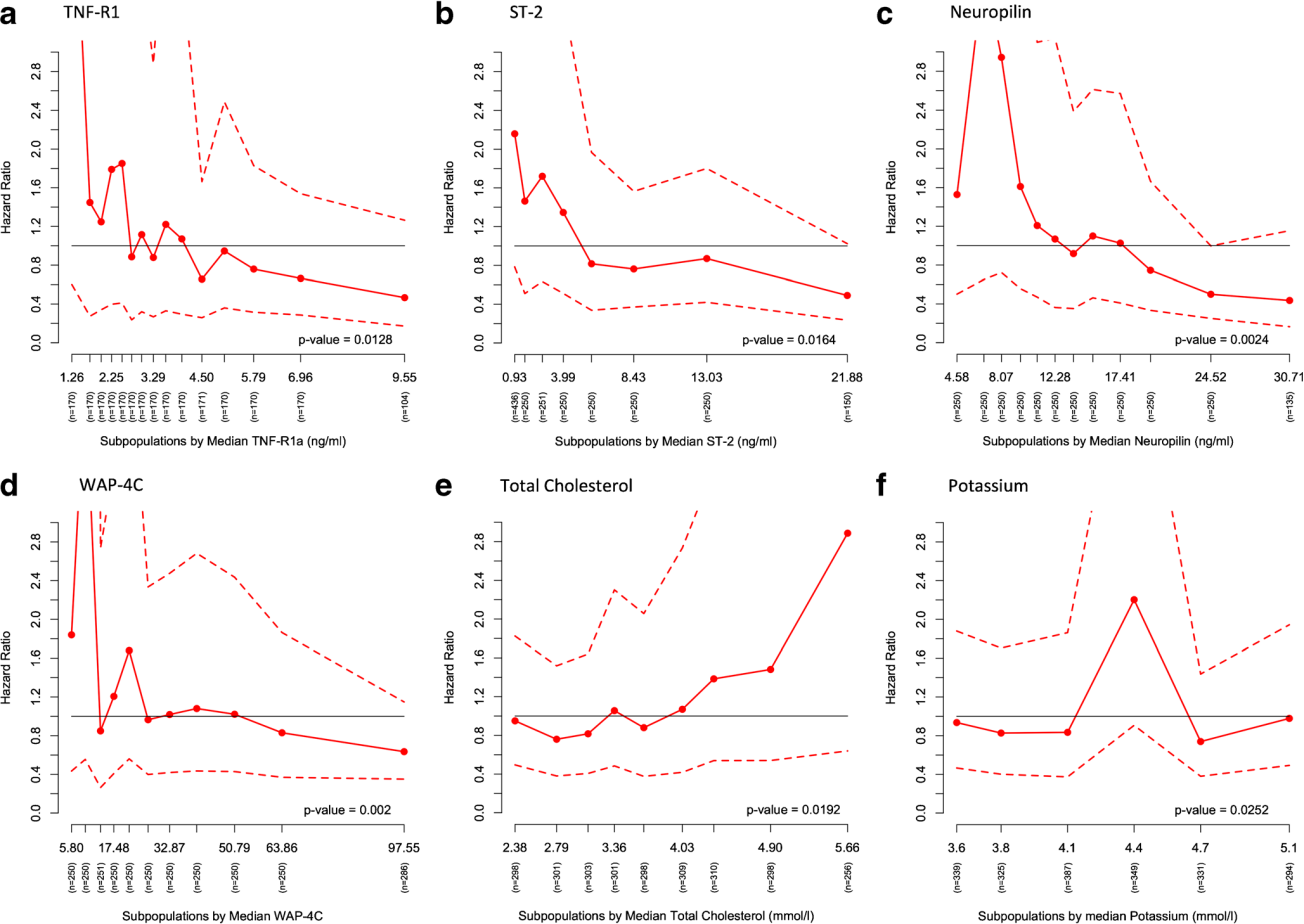
Subpopulation Treatment Effect Pattern Plot analysis of the treatment effects of rolofylline vs. placebo treatment effect measured by hazard ratio (<1 rolofylline better than placebo), with corresponding 95% confidence interval bands. *y-axis*: hazard ratio, *x-axis*: subpopulations by median. **a** *TNF-R1α*, tumor necrosis factor receptor 1 alpha levels (ng/mL) *p* value = 0.0128; **b** *ST2* levels (ng/mL) *p* value = 0.0162; **c** neuropilin levels (ng/mL) *p* value = 0.0024. **d** *WAP-4C*, WAP four-disulfide core domain protein HE4 levels (ng/mL) *p* value = 0.002; **e** Total cholesterol (mmol/L) *p* value = 0.0192. **f** Potassium (mmol/L) *p* value = 0.0252

After this, we studied the heterogeneity in treatment effect across the continuum of each biomarker. We found a divergent and consistent STEPP pattern with significant treatment interactions across the continuum of TNF-R1α (*p* = 0.013), ST-2 (*p* = 0.016), neuropilin (*p* = 0.002), WAP-4C (*p* = 0.002), total cholesterol (*p* = 0.019), and potassium (*p* = 0.025). STEPP plots for the relative risk of 180-day mortality are shown in Fig. 3. The absolute 180-day survival percentage and differences for rolofylline group and placebo group are presented in Fig. S1 of the Supplementary Material Online.

#### Identifying Responders and Nonresponders to Rolofylline

**Fig. 4:**
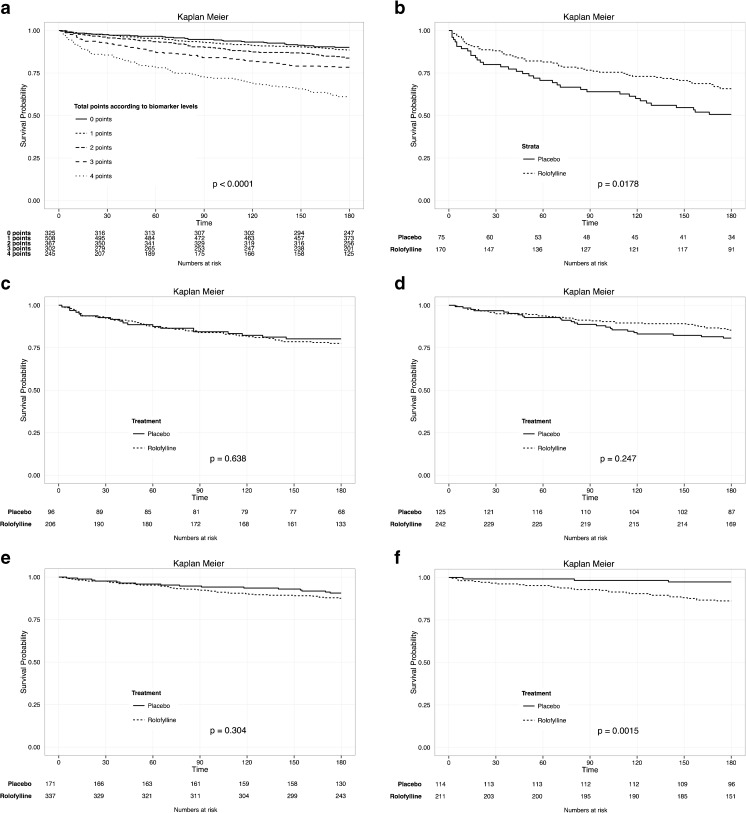
**a** Kaplan-Meier survival curves according to points subgroup. Unadjusted Kaplan-Meier survival curves according to total points based on biomarker response score chart. Log rank ≤0.0001. **b** Unadjusted Kaplan-Meier estimates of survival in patients with four points, according to treatment group. Log rank = 0.0178. **c** Unadjusted Kaplan-Meier estimates of survival in patients with three points, according to treatment group. Log rank = 0.638. **d** Unadjusted Kaplan-Meier estimates of survival in patients with two points, according to treatment group. Log rank = 0.247. **e** Unadjusted Kaplan-Meier estimates of survival in patients with one point, according to treatment. Log rank = 0.304. **f** Unadjusted Kaplan-Meier estimates of survival in patients with zero points, according to treatment. Log rank = 0.0015

The concentration where STEPP demonstrated a cross-over in relative treatment effect was considered as a clinically relevant cut-point. We dichotomized the continuous levels of these biomarkers at the level of this cut-point. According to the STEPP plots, the following thresholds were estimated: TNF-R1α: 4.00 ng/mL; ST2: 5.77 ng/mL; neuropilin 15.25 ng/mL, WAP-4C 26.92 ng/mL, total cholesterol 3.93 mmol/L, and potassium 4.1–4.7 mmol/L. The new factor variables of these dichotomized biomarkers were then entered in a multivariable Cox regression model. Multivariable Cox regression modeling yields the following *β*-coefficients: TNF-R1α 0.49, ST-2 0.48, neuropilin 0.05, WAP-4C 0.29, total cholesterol 0.45, and potassium 0.15. Neuropilin and potassium were excluded from the sum score model, due to their low *β*-coefficients. The score sum, including TNF-R1α, ST-2, WAP-4C, and total cholesterol, ranged between 0 and 4 (Table 2). In this score sum, TNF-R1α ≥ 4.00 ng/mL, ST-2 ≥ 5.77 ng/mL, and WAP-4C ≥ 26.92 ng/mL received one point, while total cholesterol ≤3.93 mmol/L received one point. Thus, TNF-R1α < 4.00 ng/mL, ST-2 < 5.77 ng/mL, and WAP-4C < 26.92 ng/mL received zero point, while total cholesterol >3.93 mmol/L received zero point. Baseline characteristics of patients per subgroup are summarized in Table S1, Supplementary Material Online. Proportion of men, age, serum creatinine, blood urea nitrogen, presence of anemia, prevalence of diabetes mellitus, smoking, ischemic heart disease, peripheral vascular disease, atrial fibrillation, device therapy, and stroke increased with increasing points. Systolic and diastolic blood pressure, heart rate, use of angiotensin converting enzyme (ACE)-inhibitors, creatinine clearance, sodium, hemoglobin, total cholesterol, and triglycerides decreased with increasing points. Kaplan-Meier analyses demonstrated that 180-day mortality increased significantly across increasing points (log-rank test *p* < 0.0001, Fig. 4a). In patients with a maximum score of four points (those with increased TNF-R1α, ST2, WAP-4C, and low total cholesterol), treatment with rolofylline was associated with improved prognosis (log-rank test *p* = 0.018), while in the intermediate score subgroups (1–3), rolofylline had no effect on outcomes. In patients with a score 0 (those with low TNF-R1α, ST2, WAP-4C, and increased total cholesterol), treatment with rolofylline was associated with worse outcome (log-rank test *p* = 0.002; Fig. 4b–f). The risk of mortality in each subgroup per point is summarized in Table 3. The treatment effect of rolofylline was then studied within each subgroup of sum score by Cox proportional hazard modeling (Table 4). In the 4-point subgroup, rolofylline significantly decreased mortality compared to placebo (HR 0.61, 95% CI 0.40–0.92, *p* = 0.019). In the 1–3-point subgroup, treatment with rolofylline did not result in differences in survival rate between the two treatment groups (1 point: HR 1.35, 95% CI 0.76–2.40, *p* = 0.306; 2 points: HR 0.74, 95% CI 0.44–1.24, *p* = 0.249; 3 points: HR 1.14, 95% CI 0.67–1.94, *p* = 0.639). In the 0-point subgroup, treatment with rolofylline was associated with worse outcome, compared to the placebo group (HR 5.52, 95% CI 1.68–18.13, *p* = 0.005). The treatment by score interaction was statistically significant (treatment by score interaction *p* < 0.001). Internal validation by subpopulation bootstrapping estimated similar hazard ratio estimates (0 points: HR 5.56, 95% CI 5.27–7–5.87; 1 point: HR 1.31, 95% CI 1.25–1.33; 2 points: HR 0.75, 95% CI 0.74–0.76; 3 points: HR 1.13, 95% CI 1.11–1.15; 4 points, HR 0.61, 95% CI 0.61–0.62) compared to the original data.

**Table 2:** Sum score for response to rolofylline

Plasma biomarker	*β*-coefficient	Points
TNF-R1α ≥4.00 ng/mL	0.49	1
ST-2 ≥5.77 ng/mL	0.48	1
WAP-4C ≥26.92 ng/mL	0.29	1
Total cholesterol ≤3.93 mmol/L	0.45	1
Total points		*4*

**Table 3:** Hazard ratio of 180-day mortality across point subgroups

Total points	Hazard ratio	95% confidence interval	*p* value
0	*Reference group*		
1	1.17	0.76–1.80	0.482
2	1.69	1.10–2.60	0.017
3	2.38	1.56–3.63	<0.001
4	4.81	3.11–7.19	<0.001

**Table 4:** The efficacy of rolofylline per point subgroups

Total points	Hazard ratio	95% confidence interval	*p* value
0	5.52	1.68–18.13	0.005
1	1.35	0.76–2.40	0.306
2	0.74	0.44–1.24	0.249
3	1.14	0.67–1.94	0.639
4	0.61	0.40–0.92	0.019

## Discussion

In the current study, we demonstrated that biomarkers may be used to identify subpopulations with distinct responses to pharmacological therapy in acute heart failure. We could not demonstrate interactions between treatment and clinical characteristics, which underscores the incremental value of biomarkers in exploring treatment heterogeneity.

Many drugs have been studied to improve clinical outcome in acute heart failure, but none of them have entered routine clinical care [2–8]. Randomized controlled studies, considered to be the golden standard to measure a treatment effect, are analyzed as a whole, and a study is considered positive if the treatment effect of the intervention group is superior to the treatment effect of the control group. However, individual differences in patient’s clinical characteristics, biomarkers, or genes may result in a differential response to treatment. This is especially the case for acute heart failure, as acute heart failure is a heterogeneous syndrome encompassing patients differing in disease severity, etiology, and comorbidities. In addition, acute heart failure patients are often treated with multiple drugs [13], which multiplies the risk of drug side effects and drug interactions. Therefore, there is a pressing need for novel approaches to study treatment heterogeneity, where the ultimate goal is to develop tools to identify groups of apparently clinically homogeneous patients but with a distinct response to acute heart failure treatment.

Subgroup analyses are necessary to identify subpopulations that differ in their response to treatment. Subgroups are often defined by clinical characteristics; however, conventional subgroup analysis based on clinical demographics often failed to demonstrate any differences in treatment effect. Circulating biomarkers however may provide more accurate information regarding related individual biological interactions influencing therapeutic response [14]. This utility would take biomarkers beyond their current role as a diagnostic or prognostic marker. A few studies have already illustrated the potential role of a single biomarker in determining drug response and treatment monitoring in heart failure [15]. ACE-inhibitors, for instance, were more effective in patients with high pre-treatment plasma renin activity [16, 17]. Also, it is hypothesized that tolvaptan may be more effective in patients with high copeptin levels, which is currently under investigation in the Acute Heart Failure Patients With High Copeptin Treated With Tolvaptan Targets Increased AVP Activation for Treatment (ACTIVATE, NCT01733134) trial.

In the current study, we used a novel approach to identify subgroups with distinct responses to rolofylline in acute heart failure. Although forest plots are generally accepted as a useful graphical method of displaying treatment effects across subgroups, this and other conventional subgroup analyses require dividing study population into spurious and incoherent subgroups, such as median or quartiles, which are not necessarily clinical relevant, and are, furthermore, associated with statistical concerns [18–21]. Also, such disjoint dividing of the study population decreases the predictive value of a continuous variable. As few patients or events may be represented in a subgroup, it may also increase the risk of false negative results, thus reducing the power to calculate the individual estimates of a treatment effect. STEPP is an exploratory graphical statistical method [10, 11] that allows studying heterogeneity of treatment across the continuous level of a covariate, in our case, a biomarker. This approach avoids the demand of dividing the study population into nonsense subgroups and allows the investigator to explore clinically relevant cut-points. In addition, STEPP analyses treatment effect in overlapping subpopulations, increases the precision of treatment estimates, making STEPP a more accurate, robust, and reliable statistical method to determine treatment heterogeneity.

In our study, we first studied differential response across clinical characteristics by forest plots. Forest plots did not reveal any interaction between clinical characteristics and rolofylline. This finding is in line with that of previous subgroup analyses of the PROTECT study population on the primary endpoint, 60-day death or cardiovascular or renal hospitalization and persistent renal impairment, showing no treatment heterogeneity across clinical characteristics [7, 9]. In contrast to clinical characteristics, we found significant interactions between treatment and several biomarkers with both forest plots and STEPP. Interestingly, we did not observe any tendency to a profile of clinical characteristics that may be associated with response to rolofylline based on the results of the forest plots, but we found a consistency in the interaction pattern of biomarkers studied by forest plots, i.e., higher levels of the majority of the measured biomarkers were related to a better treatment response to rolofylline. Since higher levels of these biomarkers indicated patients at higher risk, we suggest that rolofylline might have been more effective in patients at higher clinical risk, while it might have been harmful in the lower risk patients. Indeed, patient characteristics of each subgroup differ: patients with zero points were more likely to be younger and female and have higher blood pressure and heart rate, less diabetes and a history of ischemic heart disease, anemia, and renal dysfunction; whereas, patients with four points were more likely to be older and men and have lower blood pressures and heart rate, diabetes, ischemic heart disease, anemia, and renal dysfunction. Although we demonstrated that the responder and nonresponder subgroups differ in baseline characteristics, subgroup analyses across these characteristics did not demonstrate any interaction with treatment, emphasizing the additive value of biomarkers in studying differential reponse.

With STEPP, we were able to establish a clinically relevant cut-point, rather than to study dichotomized biomarkers at an arbitrary cut-point level (median). We developed a simple sum score to identify subgroups of patients with distinct responses to treatment. Internal validation demonstrated similar results compared to the original data. It needs to be emphasized that we were not able to perform an external validation of our results, and therefore, our results should be interpreted with caution. The present analysis was only deemed to explore the differentiating and incremental value of biomarkers in studying treatment heterogeneity. Therefore, we cannot conclude that these six biomarkers are the unique biomarker set that predicts treatment response to rolofylline. An additional interesting observation was the consistency of higher levels of the majority of the measured biomarkers to be related to a better treatment response to rolofylline. Since higher levels of these biomarkers indicated patients at higher risk, we suggest that rolofylline might have been more effective in patients with high clinical risk, while it might have been harmful in lower risk patients. This hypothesis was separately evaluated and confirmed in a recently published paper by Demissei et al. using the same cohort [22]. Baseline risk of 180-day all-cause mortality was calculated by a previously published model that combined both clinical characteristics and biomarkers [23]. In patients in low- to intermediate-risk subgroups, rolofylline was associated with a higher rate of 180-day all-cause mortality (11.9% in the rolofylline versus 8.4% in the placebo arms, *p* = 0.050). In the high-risk subgroup of patients, particularly those with estimated risk of mortality between 20 and 30%, 180-day all-cause mortality rate was markedly lower in the rolofylline arm (18.4% in the rolofylline versus 34.0% in the placebo arms, *p* = 0.003). The trend towards potential harm with rolofylline treatment in the low- to intermediate-risk subpopulations and the significant benefit in high-risk patients were also observed in the PROTECT pilot trial that served as a validation cohort [24].

The present study has several limitations. In addition to the disadvantage of post hoc analyses, our study did not include an external validation cohort, and repeated testing on the same data results in inflation of the α-levels and thereby increases the risk of finding false-positive results. Therefore, these results should be considered as explorative and should be interpreted with caution. In addition, we studied all available established and novel emerging biomarkers of the current dataset. We did not select biomarkers based on the clinical relevancy and plausible associations with the study drug.

In summary, biomarkers may be used to identify subpopulations with distinct treatment response in acute heart failure. Exploring biomarker-treatment interactions may be a selective approach to distinguish in advance those patients who may benefit from treatment from those who will most likely suffer harm without gaining benefit in acute heart failure. Future drug development programs could benefit from incorporating such an approach to match the right patient to the study drug, serving the ultimate goal to customize therapy for each individual patient.

## Electronic Supplementary Material

Fig. S1 (PNG 701 kb)

High-resolution image (TIFF 336 kb)

Table S1 (DOCX 16.7 kb)
