# Supplementary figures and images for: Identifying Subpopulations with Distinct Response to Treatment Using Plasma Biomarkers in Acute Heart Failure: Results from the PROTECT Trial: Differential Response in Acute Heart Failure
Source: Cardiovasc Drugs Ther. 2017 Jun 27;31(3):281–93. doi: 10.1007/s10557-017-6726-1 (PMC5550531; doi:10.1007/s10557-017-6726-1)

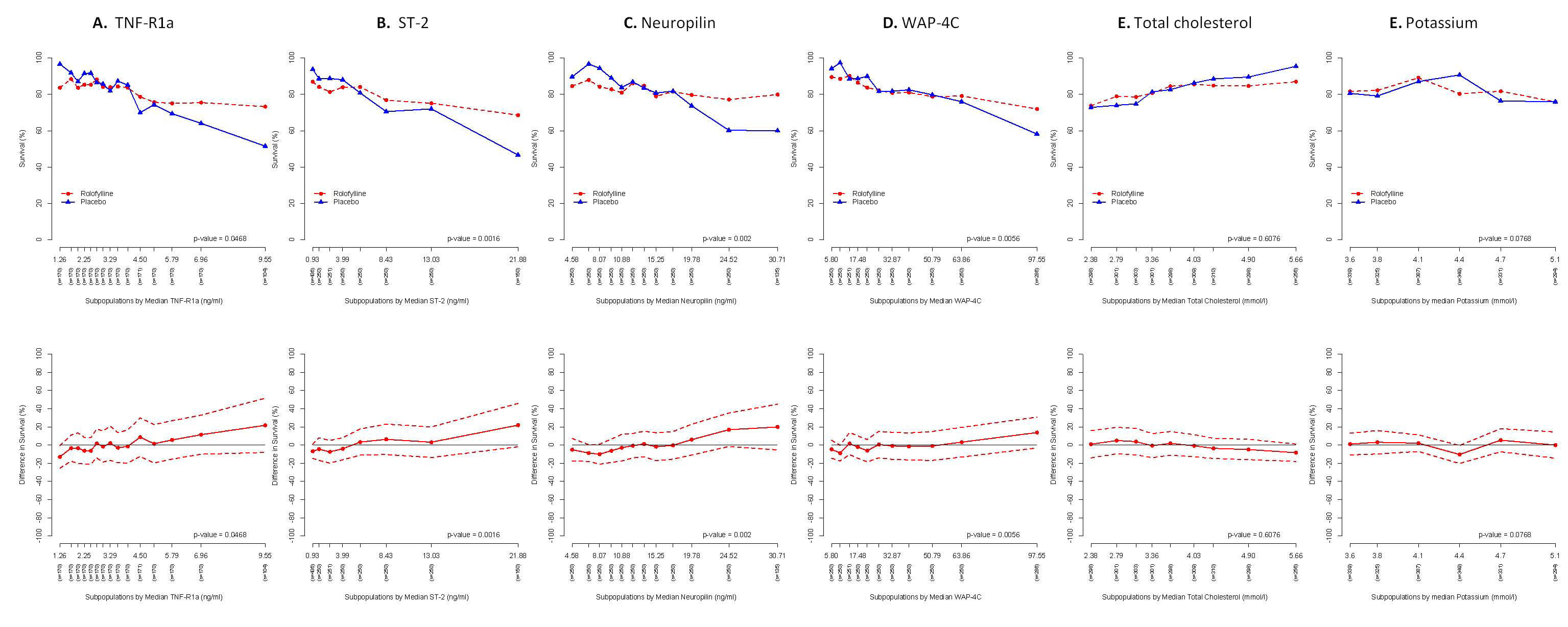

Supplement: Supplementary file 1 — (PNG 701 kb) [file 10557_2017_6726_Fig5_ESM.png]

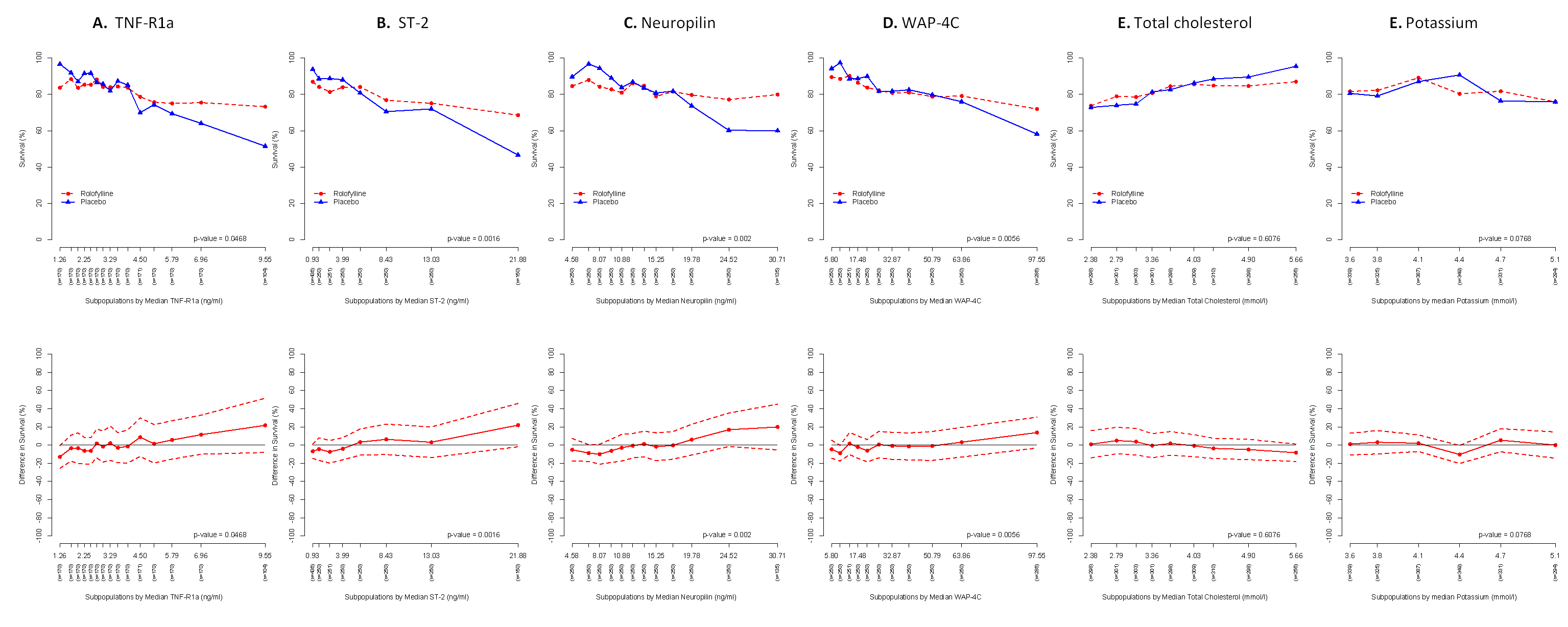

Supplement: Supplementary file 2 — High-resolution image (TIFF 336 kb) [file 10557_2017_6726_MOESM2_ESM.tif]
