# Supplementary material for: Identifying Subpopulations with Distinct Response to Treatment Using Plasma Biomarkers in Acute Heart Failure: Results from the PROTECT Trial: Differential Response in Acute Heart Failure
Source: Cardiovasc Drugs Ther. 2017 Jun 27;31(3):281–93. doi: 10.1007/s10557-017-6726-1 (PMC5550531; doi:10.1007/s10557-017-6726-1)
Supplement: Supplementary file 3 — (DOCX 16.7 kb) [file 10557_2017_6726_MOESM1_ESM.docx]

S**upplementary Material**

**Table S1: baseline characteristics according to responder-subgroup**

| **Subgroup** | **0 points** | **1 point** | **2 points** | **3 points** | **4 points** | **P for trend** |
| --- | --- | --- | --- | --- | --- | --- |
| N = | 325 | 508 | 367 | 302 | 245 |  |
| ***Demographics*** |  |  |  |  |  |  |
| Sex (% Male) | 56.9 (185) | 64.8 (329) | 66.5 (244) | 71.5 (216) | 74.7 (183) | <0.001 |
| Age (years) | 69±11.4 | 69.8±11.9 | 69.7±11.9 | 72.9±9.9 | 72.3±10.4 | <0.001 |
| BMI (kg/m2) | 28.5±5.8 | 29.1±6.4 | 28.8±6.4 | 28.6±5.6 | 28.9±5.8 | 0.901 |
| LVEF (%) | 33.3±12.6 | 32.8±13.1 | 30.5±11.8 | 32.1±13.5 | 32.1±13.6 | 0.338 |
| HFPEF (%) | 22.4 (33) | 20.2 (49) | 15 (23) | 17.7 (26) | 20 (27) | 0.424 |
| Systolic Blood Pressure (mmHg) | 129.4±15.9 | 125.6±18.2 | 124±17.3 | 122.7±17.3 | 120.5±17.4 | <0.001 |
| Diastolic Blood Pressure (mmHg) | 77.3±11.4 | 74.5±12 | 74.1±11.6 | 70.8±11.6 | 72±11.3 | <0.001 |
| Heart Rate (beats/min) | 81.9±15.6 | 81±16.1 | 80.4±15.1 | 78.1±15.3 | 79.4±15.4 | 0.006 |
| Rolofylline administration (%) | 64.9 (211) | 66.3 (337) | 65.9 (242) | 68.2 (206) | 69.4 (170) | 0.226 |
| ***Clinical Profile*** |  |  |  |  |  |  |
| Atrial fibrillation on presentaiton | 43.6 (51) | 42.7 (90) | 39.7 (56) | 42 (47) | 47.9 (46) | 0.659 |
| Orthopnea (%) | 95.7 (309) | 95.7 (472) | 97.5 (355) | 95.7 (287) | 95.9 (235) | 0.817 |
| Rales (%) | 67.1 (218) | 59.4 (301) | 63.8 (234) | 58.8 (177) | 60 (147) | 0.133 |
| Edema (%) | 58.5 (190) | 67.7 (344) | 70 (257) | 67.8 (204) | 74.3 (182) | <0.001 |
| Jugular venous pressure (%) | 36.2 (104) | 41.5 (189) | 46 (151) | 39.3 (107) | 40.8 (93) | 0.449 |
| ***Medical History*** |  |  |  |  |  |  |
| Hypertension (%) | 79.7 (259) | 81.7 (415) | 79.3 (291) | 77.2 (233) | 80 (196) | 0.469 |
| Diabetes Mellitus (%) | 36.3 (118) | 44.7 (227) | 48.5 (178) | 47 (142) | 52 (127) | <0.001 |
| Hypercholesterolemia (%) | 46.5 (151) | 49.8 (253) | 52.9 (194) | 52.2 (157) | 52.7 (129) | 0.095 |
| Smoking (%) | 17.6 (57) | 17.3 (88) | 20 (73) | 22.5 (68) | 23 (56) | 0.022 |
| Ischemic Heart Disease (%) | 65.8 (214) | 67.6 (342) | 70 (257) | 73.8 (222) | 77.1 (189) | 0.001 |
| Myocardial Infarction (%) | 45.7 (148) | 45.9 (232) | 52.6 (193) | 51.5 (155) | 56.7 (139) | 0.002 |
| PCI (%) | 16.5 (53) | 25.4 (128) | 27.7 (101) | 28 (84) | 33.2 (80) | <0.001 |
| CABG (%) | 11.5 (37) | 17.5 (88) | 22.6 (82) | 28.3 (85) | 32.4 (79) | <0.001 |
| Peripheral Vascular Disease (%) | 7.7 (25) | 9.9 (50) | 10.1 (37) | 13.9 (42) | 15.6 (38) | 0.001 |
| Atrial Fibrillation (%) | 48.6 (157) | 54.4 (276) | 53.3 (194) | 57.8 (174) | 60.7 (148) | 0.004 |
| NYHA Class |  |  |  |  |  | <0.001 |
| 1 | 20 (65) | 14.2 (72) | 15.5 (57) | 19.2 (58) | 15.9 (39) |  |
| 2 | 43.1 (140) | 49.4 (251) | 49.3 (181) | 48.7 (147) | 51.4 (126) |  |
| 3 | 32.3 (105) | 30.7 (156) | 31.1 (114) | 26.5 (80) | 28.6 (70) |  |
| ICD therapy (%) | 8.3 (27) | 14.6 (74) | 16.9 (62) | 18.6 (56) | 21.6 (53) | <0.001 |
| CRT therapy (%) | 6.8 (22) | 9.1 (46) | 12.8 (47) | 11 (33) | 13.1 (32) | 0.007 |
| Stroke (%) | 6.5 (21) | 9.3 (47) | 9.3 (34) | 10.3 (31) | 11.8 (29) | 0.033 |
| COPD (%) | 16.3 (53) | 19.3 (98) | 22.9 (84) | 20.7 (62) | 20.8 (51) | 0.127 |
| ***Prior Medication Use*** |  |  |  |  |  |  |
| ACE inhibitors or ARB (%) | 79.6 (258) | 77.2 (392) | 77.1 (283) | 70.5 (213) | 70.2 (172) | 0.001 |
| Beta blockers (%) | 72.2 (234) | 78 (396) | 75.7 (278) | 75.2 (227) | 74.7 (183) | 0.873 |
| Mineralocorticoid Receptor Antagonists (%) | 47.2 (153) | 43.7 (222) | 48.2 (177) | 40.4 (122) | 42 (103) | 0.163 |
| Calcium Antagonists (%) | 14.8 (48) | 12.4 (63) | 10.6 (39) | 16.2 (49) | 14.7 (36) | 0.561 |
| Nitrates (%) | 22.9 (74) | 23.8 (121) | 26.4 (97) | 28.5 (86) | 32.7 (80) | 0.003 |
| Digoxin (%) | 32.4 (105) | 30.3 (154) | 31.1 (114) | 25.8 (78) | 24.1 (59) | 0.013 |
| ***Laboratory Values*** |  |  |  |  |  |  |
| Creatinine (mg/dL) | 1.2 [1-1.4] | 1.2 [1-1.5] | 1.4 [1.2-1.8] | 1.6 [1.3-2.1] | 1.8 [1.4-2.2] | <0.001 |
| Creatinine Clearance (ml/min) | 59 [47.4-72.1] | 54.8 [45.3-68.6] | 46.8 [35.8-62.3] | 39.9 [31-51.1] | 37.5 [29.4-47.4] | <0.001 |
| Blood Urea Nitrogen (mg/dL) | 23 [18-29] | 25 [20-32] | 31 [24-42] | 37.5 [28.2-50] | 46 [35-59] | <0.001 |
| Sodium (mmol/L) | 141 [138-143] | 140 [138-143] | 139 [137-142] | 140 [137-142] | 138 [135-141] | <0.001 |
| Potassium (mmol/L) | 4.3 [3.9-4.7] | 4.2 [3.9-4.5] | 4.2 [3.9-4.6] | 4.3 [3.8-4.6] | 4.3 [3.8-4.7] | 0.393 |
| Hemoglobin (g/dL) | 13.5±1.8 | 12.9±1.9 | 12.5±1.9 | 12.1±2 | 12±1.9 | <0.001 |
| Anemia (%) | 22.4 (66) | 36.2 (163) | 46.9 (151) | 57.4 (152) | 59.7 (132) | <0.001 |
| Total Cholesterol (mmol/L) | 191.7±35.5 | 142±38.8 | 148.2±43.3 | 136.2±38.7 | 113.9±20.7 | <0.001 |
| Triglycerides (mmol/L) | 126.5±68.6 | 97±51.5 | 101.7±55.9 | 97.1±52.1 | 86.7±39.1 | <0.001 |
| NT-proBNP (pg/mL) | 3000 [2998.2-3000] | 3000 [3000-3539] | 3000 [3000-3489] | 3000 [3000-4232] | 3000 [3000-7283] | <0.001 |
| BNP (mg/dL) | 947 [685.5-1504.2] | 1293.8 [833-2222] | 1381 [834-2248.8] | 1430 [848-2522.4] | 1510 [1022.5-2545.2] | 0.002 |
